# Aptamer-Conjugated Multi-Quantum Dot-Embedded Silica Nanoparticles for Lateral Flow Immunoassay

**DOI:** 10.3390/bios15010054

**Published:** 2025-01-16

**Authors:** Kwanghee Yoo, Hye-Seong Cho, Jaehi Kim, Minsup Shin, Jun-Sik Chu, Sohyeon Jang, Han-Joo Bae, Heung Su Jung, Homan Kang, Bong-Hyun Jun

**Affiliations:** 1 Department of Bioscience and Biotechnology, Konkuk University, Seoul 05029, Republic of Korea; heu1997@konkuk.ac.kr (K.Y.); joh0302@konkuk.ac.kr (H.-S.C.); susia45@konkuk.ac.kr (J.K.); dnjzj159159@konkuk.ac.kr (M.S.); cjs9719@konkuk.ac.kr (J.-S.C.); thgus03030@konkuk.ac.kr (S.J.); han443811@konkuk.ac.kr (H.-J.B.); 2 Company of Global Zeus, Hwaseong 18363, Republic of Korea; hsjung@globalzeus.com; 3 Department of Radiology, Massachusetts General Hospital and Harvard Medical School, Boston, MA 02114, USA; hkang7@mgh.harvard.edu

**Keywords:** carbohydrate antigen 19-9, aptamer, quantum dot, lateral flow immunoassay

## Abstract

Lateral flow immunoassays (LFIAs) are widely used for their low cost, simplicity, and rapid results; however, enhancing their reliability requires the meticulous selection of ligands and nanoparticles (NPs). SiO_2_@QD@SiO_2_ (QD^2^) nanoparticles, which consist of quantum dots (QDs) embedded in a silica (SiO_2_) core and surrounded by an outer SiO_2_ shell, exhibit significantly higher fluorescence intensity (FI) compared to single QDs. In this study, we prepared QD^2^@PEG@Aptamer, an aptamer conjugated with QD^2^ using succinimidyl-[(N-maleimidopropionamido)-hexaethyleneglycol]ester, which is 130 times brighter than single QDs, for detecting carbohydrate antigen (CA) 19-9 through LFIA. For LFIA optimization, we determined the optimal conditions as a 1.0:2.0 × 10^−2^ ratio of polyethylene glycol (PEG) to aptamer by adjusting the amounts of PEG and aptamer, phosphate-buffered saline containing 0.5% Tween^®^ 20 as a developing solution, and 0.15 μg NPs by setting the NP weight during development. Under these conditions, QD^2^@PEG@Aptamer selectively detected CA19-9, achieving a detection limit of 1.74 × 10^−2^ mg·mL^−1^. Moreover, FI remained stable for 10 days after detection. These results highlight the potential of QD^2^ and aptamer conjugation technology as a reliable and versatile sensing platform for various diagnostic applications.

## 1. Introduction

The lateral flow immunoassay (LFIA) is a point-of-care (POC) technology, which can rapidly diagnose various diseases and detect infectious diseases at an early stage, and it has qualitative or semiquantitative characteristics [1,2,3,4,5,6]. However, LFIA has limitations, such as relatively low sensitivity that challenges the detection of analytes at low concentrations and insufficient specificity, where nonspecific ligand binding can result in false positive or false negative outcomes. These issues reduce diagnostic accuracy and restrict LFIA’s applicability in detecting critical biomarkers. The advancement of LFIA technology may enable broad applications, where the selection of ligands and design of nanoparticles (NPs) play crucial roles in achieving key advantages such as enhanced signal intensities and uniform dispersion across the strip [7,8,9]. Aptamers are single-stranded RNA or DNA that bind to specific targets, such as small molecules and proteins, with high specificity and affinity [10,11,12]. 

Aptamers offer several advantages over antibodies. As nucleic acids, they are inherently more stable and can regain their original structure even after denaturation. They exhibit high purity and strong affinity for a wide range of targets while also having low immunogenicity. Additionally, aptamers can be easily modified with functional groups to enhance their utility and can be produced at a significantly lower cost compared to antibodies [13,14,15,16,17,18]. While aptamers can be introduced into various NPs through different methods, ensuring that their activity is not lost when introducing them into NPs is important [19]. If the distance between NPs and aptamers is too small, steric hindrance may occur, thereby potentially disrupting the folding of the aptamers. To mitigate this, a spacer, such as polyethylene glycol (PEG), can increase the distance, thereby facilitating proper aptamer folding [20]. The optimal conditions for loading aptamers onto NPs must be determined based on the specific properties of each material to balance target binding affinity and prevent issues such as electrostatic repulsion or improper folding.

Sensitivity to analytes varies depending on the NP used, and recently, various detection methods have been developed based on LFIA by functionalizing various NPs [21,22,23,24]. Various types of NPs are used as probes in LFIA, including quantum dots (QDs), metal NPs, and magnetic NPs, and selecting an appropriate type based on the analytical method is crucial [25,26]. We developed QD^2^ with high quantum yield (QY), photostability, and excellent brightness [27,28,29,30,31]. These properties make QD^2^ an ideal nanoprobe for LFIA systems, with previous studies showing higher sensitivity in detecting prostate cancer and Human foreskin fibroblast (HFF) exosomes than those of conventional methods [28,29]. However, while antibodies have primarily been used as ligands in such systems, the use of aptamers remains unexplored.

Recently, studies utilizing aptamers as diagnostic tools for detecting specific targets have garnered significant interest [32]. Table 1 shows an overview of recent studies on aptamer-based LFIA systems. Aptamer-based LFIA systems have been investigated across various fields, including the detection of microorganisms [33], hormones [34], and viruses [35,36]. Among these applications, aptamers hold great potential to address the challenges of early diagnosis for pancreatic cancer, one of the most aggressive and lethal types of cancer [37,38]. Despite their numerous advantages, the number of studies focusing on aptamer-based LFIA systems remains significantly limited, and the number of aptamers successfully employed in LFIA platforms is even smaller [32]. In particular, the biomarker carbohydrate antigen (CA) 19-9, widely used for pancreatic cancer detection, has not yet been reported on in an aptamer-based fluorescence LFIA system. This highlights the need for further research and development to fully realize the potential of aptamer-based LFIA systems. 

In this study, we aimed to detect carbohydrate antigen (CA) 19-9 using QD^2^@PEG@Aptamer in an LFIA system. Specifically, we aimed to synthesize hydrophilic QD^2^ with excellent fluorescent properties and to optimize the LFIA system to detect CA19-9 without cross-reactivity with other targets.

## 2. Materials and Methods

### 2.1. Materials

Ethanol (EtOH ≥ 99.9%), tetraethyl orthosilicate (TEOS), 1-ethyl-3-(3-dimethylaminopropyl) carbodiimide hydrochloride (EDC hydrochloride), (3-aminopropyl)triethoxysilane (APTES), (3-mercaptopropyl)trimethoxysilane (MPTS), N, N-diisopropylethylamine (DIEA), bovine serum albumin (BSA), succinic anhydride, Tween^®^ 20, 2-(*N*-morpholino)ethanesulfonic hydrate (MES), prostate-specific antigen (PSA) from human semen, amyloid β 1-40 (Aβ40) rat, PrEST Antigen AFP (AFP), *N*-hydroxysulfosuccinimide (sulfo-NHS), sodium acetate trihydrate, Tris(2-carboxyethyl)phosphine hydrochloride (TCEP), and ethanolamine were purchased from Sigma-Aldrich (Gangnam-Gu, Seoul, Republic of Korea). NH_4_OH (25.0–28.0%) and N-methyl-2-pyrrolidone (NMP) were purchased from Daejung Chemical (Siheung, Gyeonggi-Do, Republic of Korea). Dichloromethane (DCM) and dimethyl sulfoxide (99.8%) were purchased from SAMCHUN Chemical (Pyeongtaek, Republic of Korea). Recombinant Human CA19-9 protein, His-tagged was purchased from Creative Biomart (Shirley, NY, USA). Amino polyethylene glycol acid (NH_2_-PEG-COOH, MW ≈ 600 Da) was purchased from Nanocs (New York, NY, USA). Recombinant human TIMP1 protein (TIMP1) was purchased from Abcam (Cambridgeshire Cambridge, England). CdSe@ZnS QDs and aptamers were purchased from Zeus (Osan, Republic of Korea). Succinimidyl-[(N-maleimidopropionamido)-hexaethyleneglycol]ester (SM(PEG)_6_) was purchased from Thermo Fisher Scientific (Waltham, MA, USA). Nitrocellulose (NC) membrane, backing card, and absorbent pad, NSE Rec. Ag (NSE), were purchased from Bore da Biotech Co., Ltd. (Seongnam, Republic of Korea). Phosphate-buffered saline (1× PBS, pH 7.4) was purchased from DYNE BIO (Seongnam, Republic of Korea). Deionized water (DIW) was obtained using direct-Q Millipore water purification system (SAM WOO S&T Co., Ltd., Seoul, Republic of Korea). Purified monoclonal antibody against CA 19-9 was purchased from BiosPacific (Emeryville, CA, USA).

### 2.2. Preparation of QD^2^

SiO_2_ NPs were synthesized using the Stöber method [47]. In brief, 40 mL EtOH, 3 mL NH_4_OH, and 1.6 mL TEOS were mixed in a 100 mL round-bottom flask at 25 °C. Then, the reaction mixture was washed several times with EtOH using centrifugation at 12,000 RCF for 10 min at 4 °C to obtain SiO_2_ NPs. Washed SiO_2_ NPs were redispersed in EtOH at 50 mg·mL^−1^. To introduce the thiol group into SiO_2_ NPs, 9.8 mL absolute EtOH, 200 μL SiO_2_ (10 mg), 100 μL DIW, 100 μL MPTS, and 25 μL NH_4_OH were taken in a 50 mL conical tube and were vigorously mixed by placing the tube within a shaking incubator for 3 h at 25 °C. After washing the solution several times with EtOH and centrifuging at 12,000 RCF for 10 min, thiol-functionalized SiO_2_ NPs were obtained. To attach CdSe@ZnS QDs to the surface of SiO_2_-SH NPs, 4 mL DCM, 200 μL SiO_2_-SH NPs (10 mg), 800 μL absolute EtOH, and 70 μL QDs (100 mg·mL^−1^) were taken in a 15 mL conical tube and incubated in a shaking incubator for 3 h at 25 °C. Then, 50 μL NH_4_OH and 50 μL MPTS were added to the solution, followed by another 3 h of incubation at 25 °C within a shaking incubator. QD-functionalized SiO_2_ NPs (SiO_2_@QDs) were collected by washing them several times with EtOH and centrifuging at 10,000 rpm for 10 min. Then, SiO_2_@QDs were dispersed in 5 mL EtOH to adjust the concentration to 2 mg·mL^−1^. To form the SiO_2_ shell, 5 mL SiO_2_@QD (2 mg·mL^−1^), 50 μL TEOS, and 50 μL NH_4_OH were incubated within a shaking incubator for 20 h. Finally, after several washes with EtOH, the solution was dispersed in 5 mL absolute EtOH to adjust the final concentration to 2 mg·mL^−1^. 

### 2.3. Conjugation of Aptamer onto QD^2^

To functionalize the QD^2^ surface with amine groups and then to introduce SM(PEG)_6_, 500 μL QD^2^ (2 mg·mL^−1^), 490 μL EtOH, 10 μL NH_4_OH, and 10 μL APTES were mixed in a microtube mixer for 1 h at 25 °C. After washing aminated QD^2^ several times with NMP, it was centrifuged at 13,000 rpm for 10 min at 4 °C and dispersed in 1 mL NMP. Next, 10 μL SM(PEG)_6_ (10 mg·mL^−1^) was added to the mixture and mixed for 2 h using a microtube mixer. Then, the mixture was washed twice with NMP and DIW each and centrifuged at 17,000 rpm for 10 min at 4. QD^2^@PEG was dispersed in 1 mL DIW. After adding 15 μg aptamer and reacting for 2 h, the resulting complex was washed twice with both PBS containing 0.5% Tween^®^ 20 (PBST) and then with 0.5% BSA in PBS, and then it was dispersed in 1 mL of 0.5% BSA in PBS. 

### 2.4. Conjugation of Anti-CA19-9 Antibody onto QD^2^

First, to aminate the silica shell of QD^2^, 500 μL QD^2^ (2 mg·mL^−1^), 10 μL NH4OH, 10 μL APTES, and 490 μL absolute EtOH were mixed in an E-tube and reacted for 1 h at 25 °C in a microtube mixer. Upon the completion of the reaction, the mixture was washed twice with NMP, and the resulting QD^2^-NH_2_ was dispersed in 500 μL NMP. To introduce carboxyl groups, 3.05 μL DIEA and 1.75 mg succinic anhydride were added to QD^2^-NH_2_, and the mixture was reacted at 25 °C for 2 h. After three washes with DIW, the product was dispersed in 700 μL DIW. Subsequently, an EDC/sulfo-NHS coupling reaction was conducted to activate the carboxyl groups before introducing PEG. In this step, 100 μL sulfo-NHS (2% *w*/*v* in DIW), 100 μL EDC hydrochloride (2% *w*/*v* in DIW), and 100 μL of 500 mM MES in DIW were added and reacted for 30 min at 25 °C. NPs were washed with 50 mM MES and dispersed in 1 mL of 50 mM MES in DIW. Then, 10 μL NH_2_-PEG-COOH (10 mg·mL^−1^) was added, and the reaction was continued for 2 h at 25 °C. The product was washed with 50 mM MES, dispersed in 1 mL of 50 mM MES in DIW, and then reacted with 3.2 μL ethanolamine at 25 °C for 30 min. After PEGylation, the product was washed thrice with DIW and dispersed in 700 μL DIW. Another EDC/sulfo-NHS coupling reaction was then performed as described. Anti-CA19-9 monoclonal antibody (A46400) was added and allowed to react for 2 h at 25 °C. Finally, the conjugates were washed twice with 0.5% PBST and twice with 0.5% BSA in PBS, and NPs were dispersed in 1 mL of 0.5% BSA in PBS.

### 2.5. Ethanol Precipitation of Aptamer

To break disulfide bonds between aptamers, 2.41 μL aptamer and 2.41 μL TCEP (100 mM) were vigorously mixed in a microtube mixer for 30 min. Then, 0.482 μL sodium acetate (3 M) and 13.26 μL absolute ETOH were added to the mixture, vortexed, and stored at −20 °C overnight. The mixture was centrifuged at 10,000 RCF for 20 min; the supernatant was removed; and 1 mL absolute EtOH was added to the pellet. It was then centrifuged at 7500 rpm for 5 min; the supernatant was removed; and the pellet was dispersed in 20 μL DIW. 

### 2.6. Characterization of SiO_2_, QDs, QD^2^, and QD^2^@PEG@Aptamer

The ultraviolet–visible (UV–Vis) absorbance of each NP was measured using a spectrophotometer (POTIZEN POP, Mecasys, Daejeon, Republic of Korea). Transmission electron microscopy–energy-dispersive X-ray spectroscopy (TEM–EDS) and transmission electron microscopy were performed using JEM-1010 and JEM-F200 (JEOL, Tokyo, Japan), respectively. Using a Cary Eclipse Fluorescence Spectrophotometer (Agilent Technologies, Santa Clara, CA, USA), the photoluminescence (PL) intensity of each NP was measured.

## 3. Results and Discussion

### 3.1. Characterization of QD^2^@PEG@Aptamer

**Figure 1:**
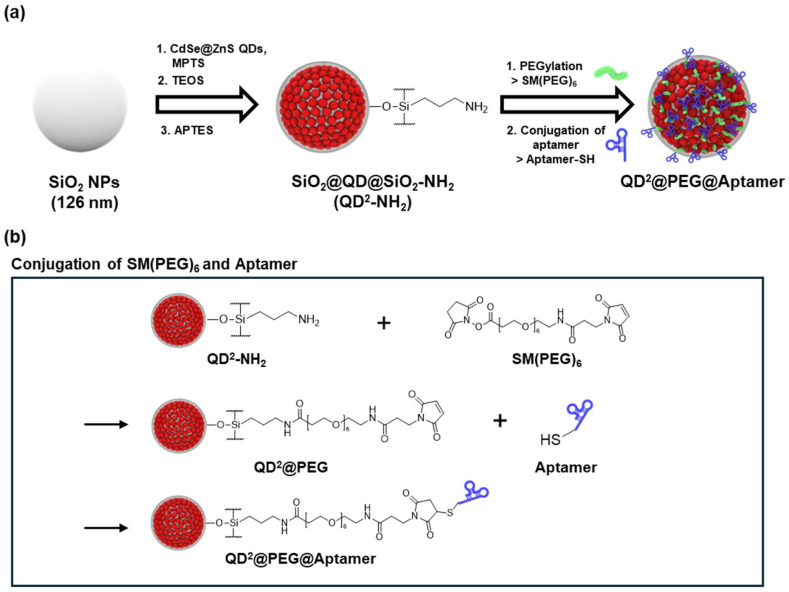
(**a**) Schematic of QD^2^@PEG@Aptamer fabrication. (**b**) Schematic of reaction process for conjugation of succinimidyl-[(N-maleimidopropionamido)-hexaethyleneglycol]ester and aptamer to QD^2^.

For LFIA applications, QD^2^, an NP consisting of multiple QDs attached to a SiO_2_ NP core with an outer SiO_2_ shell, was functionalized with PEG and an aptamer (Figure 1a) and prepared as a probe for detecting CA19-9. SiO_2_ NPs were synthesized by the Stöber method, and thiol groups were introduced onto their surface via MPTS treatment. Because of the high affinity between QDs and thiol groups, QDs were conjugated with thiolated SiO_2_ NPs (SiO_2_-SH). QD^2^ was further coated with silica, thereby providing chemical and physical stability and facilitating additional surface modifications [27]. 

An aptamer was chosen to detect CA19-9, and SM(PEG)_6_ was used as a linker to conjugate the NPs. Figure 1b illustrates the process of conjugating SM(PEG)_6_ and the aptamer to QD^2^. QD^2^ was first treated with APTES to introduce amine groups that were then linked to SM(PEG)_6_. To conjugate the aptamer with QD^2^ and prevent NP aggregation, SM(PEG)_6_ containing NHS ester and maleimide groups at both ends was used. NHS ester, known for its strong reactivity with amines, was then reacted with amine-functionalized QD^2^ (QD^2^-NH_2_) to bind with SM(PEG)_6_ [48]. The aptamer was subsequently attached to the terminus of SM(PEG)_6_ with the thiol group at the aptamer terminus by reacting to maleimide. 

**Figure 2:**
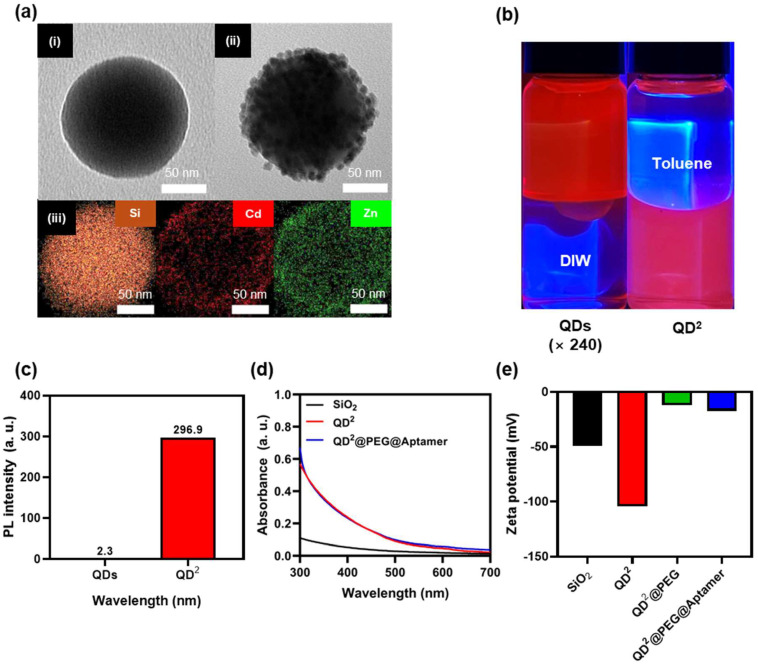
(**a**) Transmission electron microscopy–energy-dispersive X-ray spectroscopic images of (**i**) SiO_2_ and (**ii**) QD^2^ NPs, and (**iii**) energy-dispersive X-ray spectroscopic mapping images of QD^2^ (Si, Cd, and Zn) (scale bar, 50 nm). (**b**) Image showing hydrophilicity or hydrophobicity of quantum dots (QDs) and QD^2^ in vials containing equal volumes of toluene and distilled water. (**c**) Comparison of fluorescence intensities of QDs and QD^2^ under same particle concentrations (3.22 × 10^12^ particle·mL^−1^). (**d**) Ultraviolet–visible absorbance of SiO_2_, QD^2^, and QD^2^–PEG–Aptamer (50 µg·mL^−1^). (**e**) Zeta potential of NPs at each synthesis step.

QD^2^ was analyzed by TEM–EDS (Figure 2a). Silica NPs were spherical SiO_2_ NPs with a size of 126.5 nm (Figure 2a(i)). Approximately 362 QDs with a size of 6.0 nm were successfully embedded onto SiO_2_ NPs (Figure 2a(ii)). The incorporation of QDs was further confirmed through an EDS analysis that validated the presence of Cd and Zn, characteristic elements of QDs (Figure 2a(iii)). The size distributions of SiO_2_ and QD^2^, and a quantitative elemental analysis of QD^2^, are shown in Figure S1a. To confirm the successful formation of the SiO_2_ shell, QDs and QD^2^ were placed in a vial containing toluene (an organic solvent) and DIW. QDs without SiO_2_ shells were dispersed in the toluene layer, whereas QD^2^, with its hydrophilic SiO_2_ shell, was dispersed in the DIW layer, forming distinct layers (Figure 2b). We compared the PL of QDs and QD^2^ under identical NP concentrations (3.22 × 10^12^ particle·mL^−1^). The PL of QD^2^ at 620 nm was 130 times higher than that of QDs (Figure 2c). The UV–Vis measurement of SiO_2_ NPs, QD^2^, and QD^2^–aptamer showed an increase in absorbance, and no noticeable difference in the signals of QD^2^ was observed after the introduction of the aptamer (Figure 2d). The measurement of zeta potential at each stage of NP synthesis showed that the negative surface charge decreased until the QD^2^@PEG step and increased owing to the binding of the aptamer (Figure 2e) [49].

### 3.2. Optimization on LFIA Strip Based on QD^2^–Aptamer 

**Figure 3:**
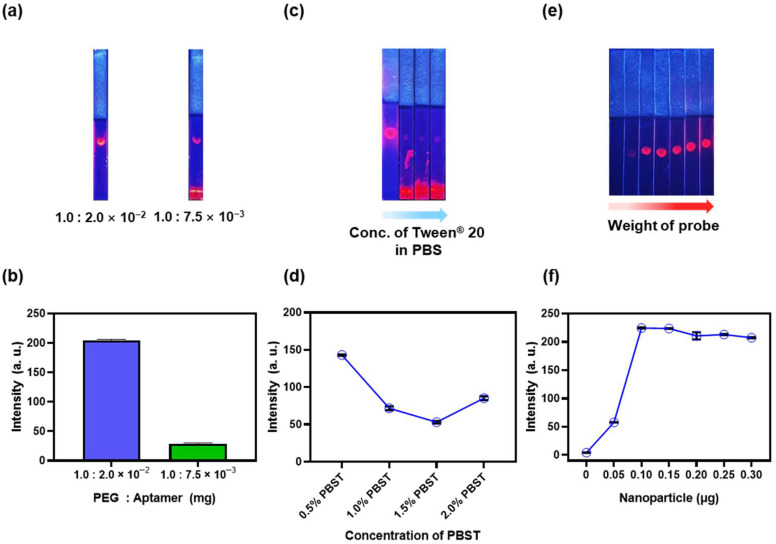
(**a**) A fluorescence image of NPs synthesized by controlling the amount of polyethylene glycol (PEG) and aptamer applied to a strip. (**b**) The fluorescence of strips with antigen at a concentration of 3 × 10^−1^ mg·mL^−1^ (PEG–aptamer). (**c**) A fluorescence image of NPs developed on a strip by adjusting the concentration of Tween^®^ 20 in phosphate-buffered saline (PBS) [0.5, 1.0, 1.5, and 2.0% Tween^®^ 20 in PBS (PBST)]. (**d**) The fluorescence intensity (FI) of strips obtained using different concentrations of Tween^®^ 20 in PBS solution (0.5, 1.0, 1.5, and 2.0% PBST). (**e**) A fluorescence image representing the optimal amount of NPs in strip development (antigen, 3 × 10^−1^ mg·mL^−1^). (**f**) The FIs of strips developed with different amounts of NPs.

CA19-9 is a biomarker of pancreatic cancer based on the Lewis antigen. CA19-9 shows the highest specificity and sensitivity for pancreatic cancer among various detectable cancers. Since a reliable biomarker for pancreatic cancer is essential, CA19-9 has been used in numerous studies on pancreatic cancer [50,51,52]. In this study, we optimized the LFIA system for detecting CA19-9 using QD^2^ conjugated with an aptamer. The LFIA strip for CA19-9 detection was prepared by dropping 0.5 μL CA19-9 antigen on an NC membrane and drying it overnight. The antigen was set at the corresponding concentration, and 30 μL of 0.5% PBST was mixed with the probe for analysis [28]. The mixture containing QD^2^@PEG@Aptamer was developed on the strip within 20 min, and this result was confirmed on the NC membrane (Figure 3a,c,e). We photographed the strips under identical conditions, analyzed the results, and separated the RGB channels with ImageJ to measure the intensity of three different areas on a strip. PEG prevents nonspecific binding and diffusion issues on NC membranes [53,54]. To effectively detect CA19-9 using an aptamer probe, we conducted experiments to establish conditions under which NPs could be properly developed on the strip with minimal nonspecific binding. Accordingly, NPs were optimized by changing the ratio of PEG to aptamer to reduce nonspecific binding and effectively develop them on the strip. The results of modifying QD^2^ with PEG-to-aptamer weight ratios of 1.0:2.0 × 10^−2^ and 1.0:7.5 × 10^−3^ are shown in Figure 3a. The NPs prepared using the first ratio exhibited significantly lower numbers of undeveloped NPs and dramatically reduced nonspecific binding than those prepared using the second ratio. No significant difference in the intensities on the NC membrane was noticed between the NPs prepared under the two conditions (Figure 3b). Still, several NPs were not undeveloped while using the second ratio, and the background noise was relatively high. Tween^®^ 20, a commonly used surfactant and detergent in LFIA, primarily reduces nonspecific binding and regulates the flow rate of NPs through a pad [55,56]. We further optimized LFIA by adjusting the concentration of Tween^®^ 20 in PBS. The concentrations of Tween^®^ 20 in PBS at 0.5, 1.0, 1.5, and 2.0% were used to evaluate its effect on the performance of LFIA. As the concentration of Tween^®^ 20 increased, the flow rate of the buffer across the LFIA strip increased, preventing NPs from fully developing on the membrane (Figure 3c). This indicates that relatively high concentrations of Tween^®^ 20 can hinder optimal NP interaction and signal development by accelerating the flow, thereby leading to incomplete test results. The FIs of each condition were measured, and FI was found to be the highest at 0.5% Tween^®^ 20 concentration when NPs were entirely developed (Figure 3d). We developed different amounts of probe on each strip with the same concentration of antigen (Figure 3e). FI was saturated at 0.10 µg, and the signal did not increase further (Figure 3f). Based on these results, 0.15 µg NPs, which showed the lowest standard deviation and highest FI, were selected for the subsequent experiments.

### 3.3. Validation of CA19-9 Detection Performance, Specificity, and Photostability Using QD^2^@PEG@Aptamer-Based LFIA

**Figure 4:**
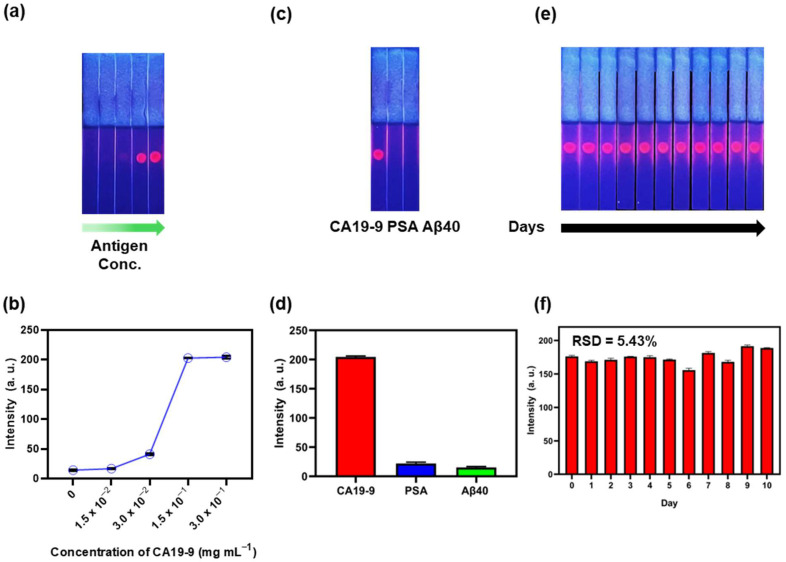
(**a**) Fluorescence image of strip with developed QD^2^–PEG–Aptamer containing different concentrations of antigen (0, 1.5 × 10^−2^, 3.0 × 10^−2^, 1.5 × 10^−1^, and 3.0 × 10^−1^ mg·mL^−1^). (**b**) FIs measured on strip detecting CA19-9 using QD^2^–PEG–Aptamer. (**c**) Fluorescence images of NPs developed on different strips after drying CA19-9, PSA, and amyloid beta 1-40 (Aβ40) to determine selectivity of aptamer. (**d**) FIs measured on strips with CA19-9, PSA, and Aβ40. (**e**) Strip image showing antigen detected at concentration of 3 × 10^−1^ mg·mL^−1^ on test paper over 10-day period (days 0–10). (**f**) FIs measured from strips photographed over 10-day period (days 0–10).

To verify the performance of the aptamer, we used four different concentrations of the antigen: 1.5 × 10^−2^, 3.0 × 10^−2^, 1.5 × 10^−1^, and 3.0 × 10^−1^ mg·mL^−1^. Detection using the aptamer was visually confirmed up to the 3.0 × 10^−2^ mg·mL^−1^ antigen concentration (Figure 4a). FIs measured in three different areas of the same strip were 204.3, 202.8, and 41.2 at antigen concentrations of 3.0 × 10^−1^, 1.5 × 10^−1^, and 3.0 × 10^−2^ mg·mL^−1^ (Figure 4b). In addition, fluorescence signals were similar regardless of the area of detection. When CA19-9 was measured using the corresponding antibody under the same conditions, detection was possible up to the same concentration as that observed with the aptamer. FI was also identical (Figure S2a). The FI graphs for the aptamer and antibody showed almost similar patterns (Figure S2b). Figure S2c shows the limit of detection (LOD) values measured at various concentrations of CA19-9 using the aptamer and antibody. Both methods resulted in LOD values of 1.74 × 10^−2^ mg·mL^−1^, with an R^2^ value of 0.99, thereby indicating high reliability. A selectivity experiment was conducted to test whether the aptamer selectively detected only CA19-9. Aβ40, a biomarker for Alzheimer’s disease, and PSA, a biomarker for prostate cancer, were dropped onto each strip and dried, and then the NPs were developed (Figure 4c). Only the strip containing CA19-9 showed FI, and other antigens were not detected. The FIs of each strip were 204.5, 22.0, and 15.2 for CA19-9, Aβ40, and PSA, respectively (Figure 4d), indicating that the aptamer selectively detected CA19-9. To further investigate whether QD^2^@PEG@Aptamer exhibits nonspecific binding to other antigens, we tested its specificity against various biomarkers, including amyloid beta-40 (Aβ40), a biomarker for Alzheimer’s disease; tissue inhibitor of metalloproteinase 1 (TIMP1), a biomarker for pancreatic cancer; alpha-fetoprotein (AFP), a biomarker for ovarian cancer; and neuron-specific enolase (NSE), a biomarker for lung cancer. Each biomarker was dried onto the strip at a uniform concentration of 0.15 mg·mL^−1^, and QD^2^@PEG@Aptamer was developed across the strip (Figure S3a). The FIs measured for CA19-9, Aβ40, TIMP1, AFP, and NSE were 202.83, 5.33, 5.00, 6.66, and 2.66, respectively. Among these, only CA19-9 produced a detectable fluorescence signal, while all other biomarkers showed negligible signals (Figure S3b). These results confirm that QD^2^@PEG@Aptamer specifically detects CA19-9 without nonspecific interactions with other biomarkers. To assess the photostability of the strip detecting CA19-9, photographs were captured daily for 10 days (Figure 4e). FIs were found to be constant owing to the low photobleaching property of QD^2^ for 10 days. Figure 4f shows FIs, and the relative standard deviation (RSD) value was as low as 5.43%, indicating that FI did not significantly decrease even after a long period of time. Overall, these results demonstrated that the synthesized probe successfully detected CA19-9 via the aptamer that selectively bound to CA19-9 and maintained FI over an extended time period.

## 4. Conclusions

We successfully synthesized QD^2^@PEG@Aptamer by selecting QD^2^ as the probe and an aptamer as the ligand for detecting CA19-9 in an LFIA system. Our results demonstrated the reliability and versatility of the LFIA system using QD^2^@PEG@Aptamer for CA19-9 detection. This approach shows excellent potential for detecting various biomarkers with high sensitivity, selectivity, and diagnostic reliability. 

## Figures and Tables

**Table 1:** Representative research status of detecting various targets using aptamer-based LFIA system.

Reporter	Target	Assay Type	Limit of Detection	Reference
Gold nanoparticle	SARS-CoV-2	cLFIA	91.2 ng·mL^−1^	[39]
	Dopamine	cLFIA	50 ng·mL^−1^	[40]
Quantum dot	Ochratoxin A	fLFIA	1.9 ng·mL^−1^	[41]
	*Salmonella* Typhimurium	fLFIA	10^3^ cfu·mL^−1^	[42]
Upconversion nanoparticle	Vaspin	fLFIA	39 pg·mL^−1^	[43]
	*Salmonella*	fLFIA	85 cfu·mL^−1^	[44]
Magnetic nanoparticle	Kanamycin	HPLC	4.96 nM	[45]
	*L. monocytogenes*	fLFIA	1.0 × 10^2^ cfu·mL^−1^	[46]

## Data Availability

Data are contained within this article or the Supplementary Materials.

## Supplementary Materials

The following supporting information can be downloaded at https://www.mdpi.com/article/10.3390/bios15010054/s1, Figure S1: (a) Size distribution of SiO_2_ and QD^2^. (b) Energy-dispersive spectroscopic elemental mapping images of Si, O, Cd, Se, Zn, and S; Figure S2: (a) (i) An image of a strip image detecting the CA19-9 antigen at various concentrations using an antibody. (a) (ii) FI measured on strip detecting CA19-9 using QD^2^@PEG@Antibody. (b) FIs of QD^2^–PEG–Aptamer and QD^2^–PEG–Antibody at each CA19-9 concentration. (c) (i) Graph showing limit of detection (LOD) of QD^2^@PEG@Aptamer at various CA19-9 concentrations. (ii) Graph showing LOD of QD^2^@PEG@Antibody at various CA19-9 concentrations; Figure S3: (a) Fluorescence images of QD^2^–PEG–Aptamer developed on different strips after drying Aβ40, TIMP1, AFP, and NSE to determine nonspecific binding of aptamer; (b) FI measured on strips with Aβ40, TIMP1, AFP, and NSE.

## References

[1] Mahmoudi T., de la Guardia M., Baradaran B. (2020). Lateral flow assays towards point-of-care cancer detection: A review of current progress and future trends. TrAC Trends Anal. Chem..

[2] Shin M., Kim W., Yoo K., Cho H.-S., Jang S., Bae H.-J., An J., Lee J.-C., Chang H., Kim D.-E. (2024). Highly sensitive multiplexed colorimetric lateral flow immunoassay by plasmon-controlled metal–silica isoform nanocomposites: PINs. Nano Converg..

[3] Liu S., Liao Y., Shu R., Sun J., Zhang D., Zhang W., Wang J. (2024). Evaluation of the Multidimensional Enhanced Lateral Flow Immunoassay in Point-of-Care Nanosensors. ACS Nano.

[4] Kakkar S., Gupta P., Yadav S.P.S., Raj D., Singh G., Chauhan S., Mishra M.K., Martín-Ortega E., Chiussi S., Kant K. (2024). Lateral flow assays: Progress and evolution of recent trends in point-of-care applications. Mater. Today Bio.

[5] Kim K., Kashefi-Kheyrabadi L., Joung Y., Kim K., Dang H., Chavan S.G., Lee M.-H., Choo J. (2021). Recent advances in sensitive surface-enhanced Raman scattering-based lateral flow assay platforms for point-of-care diagnostics of infectious diseases. Sens. Actuators B Chem..

[6] Kim J., Shin M.-S., Shin J., Kim H.-M., Pham X.-H., Park S.-M., Kim D.-E., Kim Y.J., Jun B.-H. (2023). Recent trends in lateral flow immunoassays with optical nanoparticles. Int. J. Mol. Sci..

[7] Sena-Torralba A., Álvarez-Diduk R., Parolo C., Piper A., Merkoçi A. (2022). Toward next generation lateral flow assays: Integration of nanomaterials. Chem. Rev..

[8] Nguyen V.-T., Song S., Park S., Joo C. (2020). Recent advances in high-sensitivity detection methods for paper-based lateral-flow assay. Biosens. Bioelectron..

[9] Lou D., Fan L., Jiang T., Zhang Y. (2022). Advances in nanoparticle-based lateral flow immunoassay for point-of-care testing. View.

[10] Xiao X., Li H., Zhao L., Zhang Y., Liu Z. (2021). Oligonucleotide aptamers: Recent advances in their screening, molecular conformation and therapeutic applications. Biomed. Pharmacother..

[11] Ning Y., Hu J., Lu F. (2020). Aptamers used for biosensors and targeted therapy. Biomed. Pharmacother..

[12] Zhu C., Feng Z., Qin H., Chen L., Yan M., Li L., Qu F. (2024). Recent progress of SELEX methods for screening nucleic acid aptamers. Talanta.

[13] Darmostuk M., Rimpelova S., Gbelcova H., Ruml T. (2015). Current approaches in SELEX: An update to aptamer selection technology. Biotechnol. Adv..

[14] Liu Y., Deng Y., Li T., Chen Z., Chen H., Li S., Liu H. (2018). Aptamer-based electrochemical biosensor for mercury ions detection using AuNPs-modified glass carbon electrode. J. Biomed. Nanotechnol..

[15] Zi-Jian W., Er-Ning C., Ge Y., Xin-Ying Z., Feng Q. (2020). Research advances of aptamers selection for small molecule targets. Chin. J. Anal. Chem..

[16] Zhongcheng L., Lijian Z., Lipeng L., Yanfen Z., Nannan W., Yao C., Xianghuan W. (2015). Novel method based on colloidal gold for detection of oligonucleotide aptamer with protein interacting. Chem. J. Chin. Univ. Chin..

[17] Woo H.-M., Kim K.-S., Lee J.-M., Shim H.-S., Cho S.-J., Lee W.-K., Ko H.W., Keum Y.-S., Kim S.-Y., Pathinayake P. (2013). Single-stranded DNA aptamer that specifically binds to the influenza virus NS1 protein suppresses interferon antagonism. Antivir. Res..

[18] Zhong Y., Zhao J., Li J., Liao X., Chen F. (2020). Advances of aptamers screened by Cell-SELEX in selection procedure, cancer diagnostics and therapeutics. Anal. Biochem..

[19] Urmann K., Modrejewski J., Scheper T., Walter J.-G. (2017). Aptamer-modified nanomaterials: Principles and applications. BioNanoMaterials.

[20] Zhu G., Lübbecke M., Walter J.G., Stahl F., Scheper T. (2011). Characterization of optimal aptamer-microarray binding chemistry and spacer design. Chem. Eng. Technol..

[21] Kim Y.J., Rho W.-Y., Park S.-M., Jun B.-H. (2024). Optical nanomaterial-based detection of biomarkers in liquid biopsy. J. Hematol. Oncol..

[22] Yang H., He Q., Lin M., Ji L., Zhang L., Xiao H., Li S., Li Q., Cui X., Zhao S. (2022). Multifunctional Au@ Pt@ Ag NPs with color-photothermal-Raman properties for multimodal lateral flow immunoassay. J. Hazard. Mater..

[23] Zhao T., Liang P., Ren J., Zhu J., Yang X., Bian H., Li J., Cui X., Fu C., Xing J. (2023). Gold-silver alloy hollow nanoshells-based lateral flow immunoassay for colorimetric, photothermal, and SERS tri-mode detection of SARS-CoV-2 neutralizing antibody. Anal. Chim. Acta.

[24] Guo G., Zhao T., Sun R., Song M., Liu H., Wang S., Li J., Zeng J. (2024). Au-Fe_3_O_4_ dumbbell-like nanoparticles based lateral flow immunoassay for colorimetric and photothermal dual-mode detection of SARS-CoV-2 spike protein. Chin. Chem. Lett..

[25] Wang Y., Xu H., Wei M., Gu H., Xu Q., Zhu W. (2009). Study of superparamagnetic nanoparticles as labels in the quantitative lateral flow immunoassay. Mater. Sci. Eng. C.

[26] Berlina A.N., Taranova N.A., Zherdev A.V., Vengerov Y.Y., Dzantiev B.B. (2013). Quantum dot-based lateral flow immunoassay for detection of chloramphenicol in milk. Anal. Bioanal. Chem..

[27] Jun B.H., Hwang D.W., Jung H.S., Jang J., Kim H., Kang H., Kang T., Kyeong S., Lee H., Jeong D.H. (2012). Ultrasensitive, Biocompatible, Quantum-Dot-Embedded Silica Nanoparticles for Bioimaging. Adv. Funct. Mater..

[28] Bock S., Kim H.-M., Kim J., An J., Choi Y.-S., Pham X.-H., Jo A., Ham K.-m., Song H., Kim J.-W. (2021). Lateral flow immunoassay with quantum-dot-embedded silica nanoparticles for prostate-specific antigen detection. Nanomaterials.

[29] Kim H.-M., Oh C., An J., Baek S., Bock S., Kim J., Jung H.-S., Song H., Kim J.-W., Jo A. (2021). Multi-quantum dots-embedded silica-encapsulated nanoparticle-based lateral flow assay for highly sensitive exosome detection. Nanomaterials.

[30] Ham K.-M., Kim M., Bock S., Kim J., Kim W., Jung H.S., An J., Song H., Kim J.-W., Kim H.-M. (2022). Highly bright silica-coated InP/ZnS quantum dot-embedded silica nanoparticles as biocompatible nanoprobes. Int. J. Mol. Sci..

[31] Pham X.-H., Park S.-M., Ham K.-M., Kyeong S., Son B.S., Kim J., Hahm E., Kim Y.-H., Bock S., Kim W. (2021). Synthesis and application of silica-coated quantum dots in biomedicine. Int. J. Mol. Sci..

[32] Majdinasab M., Badea M., Marty J.L. (2022). Aptamer-Based lateral flow assays: Current trends in clinical diagnostic rapid tests. Pharmaceuticals.

[33] Ren Y., Gao P., Song Y., Yang X., Yang T., Chen S., Fu S., Qin X., Shao M., Man C. (2021). An aptamer-exonuclease III (Exo III)–assisted amplification-based lateral flow assay for sensitive detection of Escherichia coli O157: H7 in milk. J. Dairy Sci..

[34] Dalirirad S., Steckl A.J. (2019). Aptamer-based lateral flow assay for point of care cortisol detection in sweat. Sens. Actuators B Chem..

[35] Zhou Y., Wu Y., Ding L., Huang X., Xiong Y. (2021). Point-of-care COVID-19 diagnostics powered by lateral flow assay. TrAC Trends Anal. Chem..

[36] Le T.T., Chang P., Benton D.J., McCauley J.W., Iqbal M., Cass A.E. (2017). Dual recognition element lateral flow assay toward multiplex strain specific influenza virus detection. Anal. Chem..

[37] Kaur H., Bruno J.G., Kumar A., Sharma T.K. (2018). Aptamers in the therapeutics and diagnostics pipelines. Theranostics.

[38] Li Q., Maier S., Li P., Peterhansl J., Belka C., Mayerle J., Mahajan U.M. (2020). Aptamers: A novel targeted theranostic platform for pancreatic ductal adenocarcinoma. Radiat. Oncol..

[39] Li X., Wang J., Yang G., Fang X., Zhao L., Luo Z., Dong Y. (2024). The Development of Aptamer-Based Gold Nanoparticle Lateral Flow Test Strips for the Detection of SARS-CoV-2 S Proteins on the Surface of Cold-Chain Food Packaging. Molecules.

[40] Dalirirad S., Steckl A.J. (2020). Lateral flow assay using aptamer-based sensing for on-site detection of dopamine in urine. Anal. Biochem..

[41] Wang L., Chen W., Ma W., Liu L., Ma W., Zhao Y., Zhu Y., Xu L., Kuang H., Xu C. (2011). Fluorescent strip sensor for rapid determination of toxins. Chem. Commun..

[42] Shang Y., Cai S., Ye Q., Wu Q., Shao Y., Qu X., Xiang X., Zhou B., Ding Y., Chen M. (2022). Quantum dot nanobeads-labelled lateral flow immunoassay strip for rapid and sensitive detection of *Salmonella* Typhimurium based on strand displacement loop-mediated isothermal amplification. Engineering.

[43] Ali M., Sajid M., Khalid M.A.U., Kim S.W., Lim J.H., Huh D., Choi K.H. (2020). A fluorescent lateral flow biosensor for the quantitative detection of Vaspin using upconverting nanoparticles. Spectrochim. Acta Part A Mol. Biomol. Spectrosc..

[44] Jin B., Yang Y., He R., Park Y.I., Lee A., Bai D., Li F., Lu T.J., Xu F., Lin M. (2018). Lateral flow aptamer assay integrated smartphone-based portable device for simultaneous detection of multiple targets using upconversion nanoparticles. Sens. Actuators B Chem..

[45] Ou Y., Jin X., Liu J., Tian Y., Zhou N. (2019). Visual detection of kanamycin with DNA-functionalized gold nanoparticles probe in aptamer-based strip biosensor. Anal. Biochem..

[46] Du J., Liu J., Liu K., Zhao D., Sagratini G., Tao J., Bai Y. (2022). Development of a fluorescent test strip sensor based on surface positively-charged magnetic bead separation for the detection of Listeria monocytogenes. Anal. Methods.

[47] Stöber W., Fink A., Bohn E. (1968). Controlled growth of monodisperse silica spheres in the micron size range. J. Colloid Interface Sci..

[48] del Pino M.M.S. (2011). Simple chemical tools to expand the range of proteomics applications. J. Proteom..

[49] Xiao Y., Lin L., Shen M., Shi X. (2019). Design of DNA aptamer-functionalized magnetic short nanofibers for efficient capture and release of circulating tumor cells. Bioconjugate Chem..

[50] Luo G., Guo M., Jin K., Liu Z., Liu C., Cheng H., Lu Y., Long J., Liu L., Xu J. (2016). Optimize CA19-9 in detecting pancreatic cancer by Lewis and Secretor genotyping. Pancreatology.

[51] Kannagi R. (2007). Carbohydrate antigen sialyl Lewis a-its pathophysiological significance and induction mechanism in cancer progression. Chang. Gung Med. J..

[52] Luo G., Liu C., Guo M., Cheng H., Lu Y., Jin K., Liu L., Long J., Xu J., Lu R. (2017). Potential biomarkers in Lewis negative patients with pancreatic cancer. Ann. Surg..

[53] Javani A., Javadi-Zarnaghi F., Rasaee M.J. (2017). Development of a colorimetric nucleic acid-based lateral flow assay with non-biotinylated capture DNA. Appl. Biol. Chem..

[54] Sheng J.C.-C., De La Franier B., Thompson M. (2021). Assembling surface linker chemistry with minimization of non-specific adsorption on biosensor materials. Materials.

[55] Wu Y., Hu Y., Jiang N., Anantharanjit R., Yetisen A.K., Cordeiro M.F. (2022). Quantitative brain-derived neurotrophic factor lateral flow assay for point-of-care detection of glaucoma. Lab A Chip.

[56] Parolo C., Sena-Torralba A., Bergua J.F., Calucho E., Fuentes-Chust C., Hu L., Rivas L., Álvarez-Diduk R., Nguyen E.P., Cinti S. (2020). Tutorial: Design and fabrication of nanoparticle-based lateral-flow immunoassays. Nat. Protoc..

