# Supplementary material for: Aptamer-Conjugated Multi-Quantum Dot-Embedded Silica Nanoparticles for Lateral Flow Immunoassay
Source: Biosensors (Basel). 2025 Jan 16;15(1):54. doi: 10.3390/bios15010054 (PMC11763673; doi:10.3390/bios15010054)
Supplement: Supplementary file 1 [file biosensors-15-00054-s001.zip › Supplementary_Materials.pdf]

Supplementary Materials

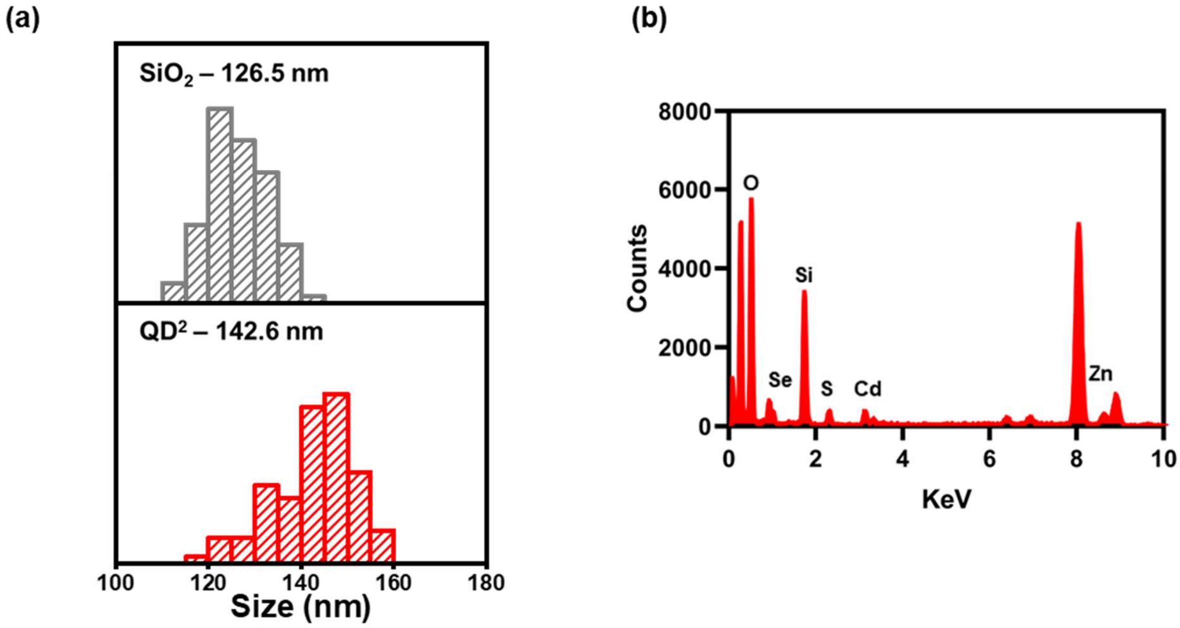

Figure S1. (a) Size distribution of SiO_2 and QD^2. (b) Energy dispersive spectroscopic elemental mapping images of Si, O, Cd, Se, Zn, and S.

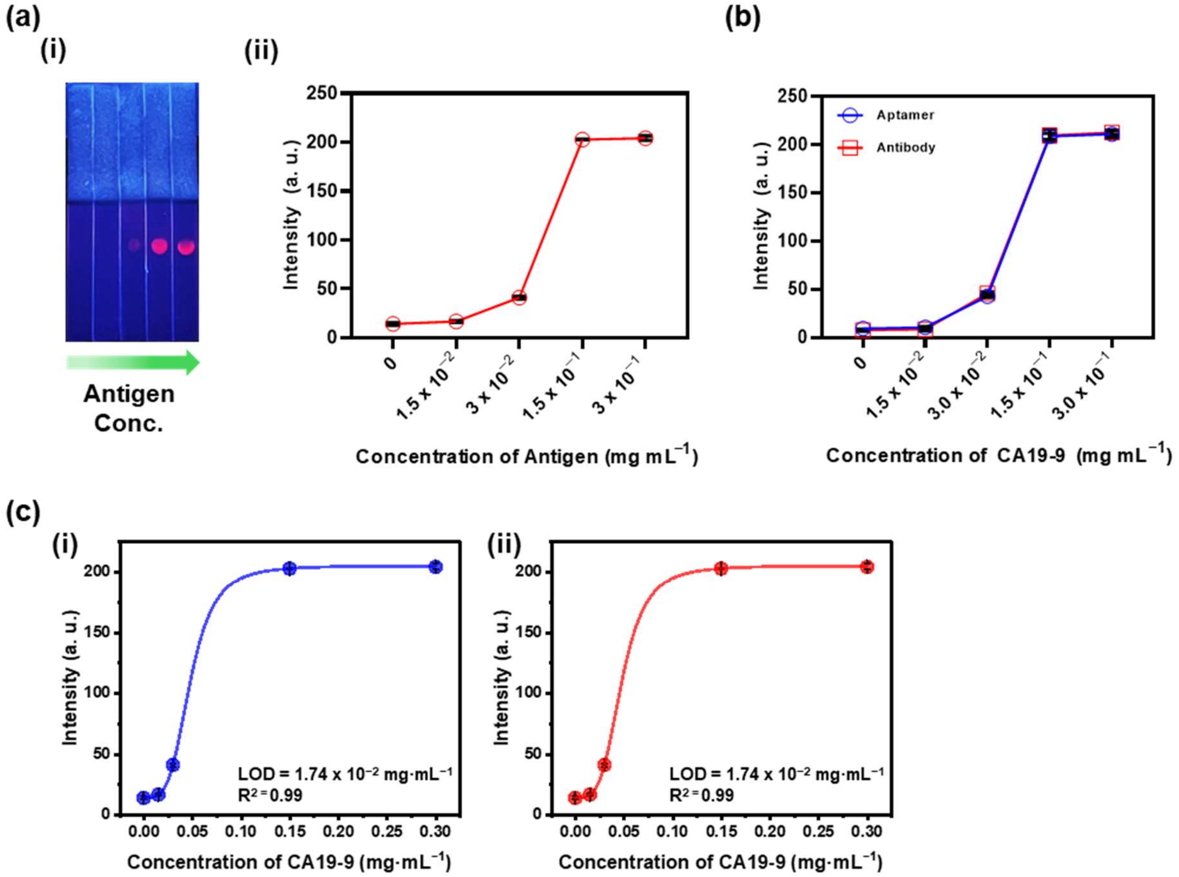

Figure S2. (a) (i) An image of a strip image detecting CA19-9 antigen at various concentrations using antibody. (a) (ii) FI measured on a strip detecting CA19-9 using QD^2@PEG@Antibody. (b) FIs of QD^2-PEG-Aptamer and QD^2-PEG-Antibody at each CA19-9 concentration. (c) (i) Graph showing limit of detection (LOD) of QD^2@PEG@Aptamer at various CA19-9 concentrations. (ii) Graph showing LOD of QD^2@PEG@Antibody at various CA19-9 concentrations.

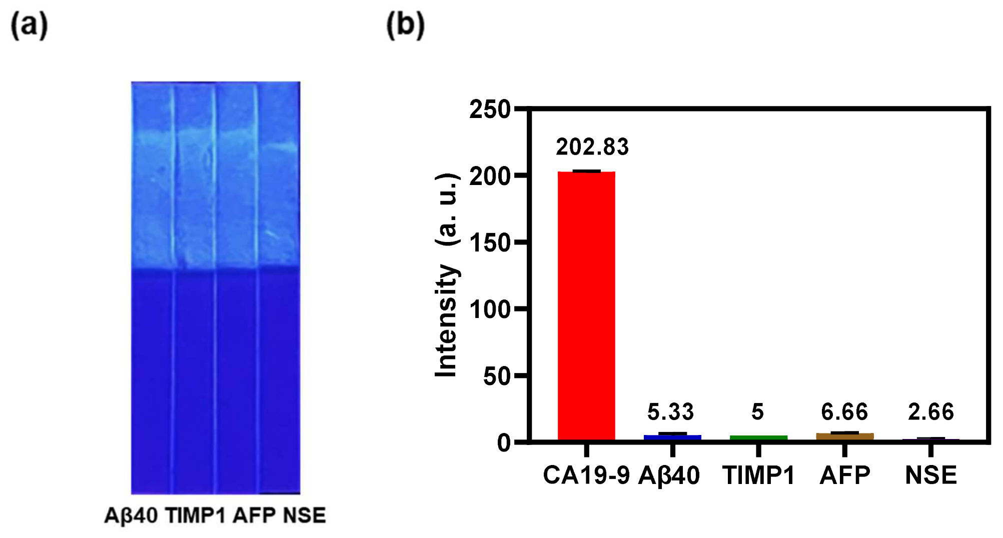

Figure S3. (a) Fluorescence images of QD^2@PEG@Aptamer developed on different strips after drying Aβ40, TIMP1, AFP, and NSE to determine the non-specific binding of the aptamer; (b) FI measured on strips with Aβ40, TIMP1, AFP, and NSE.
